# Coastal Fisheries in the Eastern Baltic Sea (Gulf of Finland) and Its Basin from the 15 to the Early 20^th^ Centuries

**DOI:** 10.1371/journal.pone.0077059

**Published:** 2013-10-24

**Authors:** Julia Lajus, Alexei Kraikovski, Dmitry Lajus

**Affiliations:** 1 National Research University Higher School of Economics, St. Petersburg, Russia; 2 European University at St. Petersburg, St. Petersburg, Russia; 3 St. Petersburg State University, St. Petersburg, Russia; Leibniz Center for Tropical Marine Ecology, Germany

## Abstract

The paper describes and analyzes original data, extracted from historical documents and scientific surveys, related to Russian fisheries in the southeastern part of the Gulf of Finland and its inflowing rivers during the 15- early 20^th^ centuries. The data allow tracing key trends in fisheries development and in the abundance of major commercial species. In particular, results showed that, over time, the main fishing areas moved from the middle part of rivers downstream towards and onto the coastal sea. Changes in fishing patterns were closely interrelated with changes in the abundance of exploited fish. Anadromous species, such as Atlantic sturgeon, Atlantic salmon, brown trout, whitefish, vimba bream, smelt, lamprey, and catadromous eel were the most important commercial fish in the area because they were abundant, had high commercial value and were easily available for fishing in rivers. Due to intensive exploitation and other human-induced factors, populations of most of these species had declined notably by the early 20^th^ century and have now lost commercial significance. The last sturgeon was caught in 1996, and today only smelt and lamprey support small commercial fisheries. According to historical sources, catches of freshwater species such as roach, ide, pike, perch, ruffe and burbot regularly occurred, in some areas exceeding half of the total catch, but they were not as important as migrating fish and no clear trends in abundance are apparent. Of documented marine catch, Baltic herring appeared in the 16^th^ century, but did not become commercially significant until the 19^th^ century. From then until now herring have been the dominant catch.

## Introduction

The study of fisheries history may provide information that is key to understanding the history of aquatic ecosystems and to identifying and quantifying factors that affect populations of particular species [1], [2] Fisheries data usually appear much earlier than other ecosystem data. Thus, they allow us to examine conditions that predate global changes caused by human activities, and also reveal centennial-scale fluctuations in biota. Such information may be useful for ecosystem management because it allows to estimate carrying capacity of ecosystems in times when anthropogenic pressure was weaker than now, and to obtain information on the historical distribution of species. Historical data are often inconsistent, which causes serious difficulties when applying formal analytical methods, in particular, statistics. Due to this such data are not always fully taken into account. Ignoring anecdotal historical data may result in the “shifting baseline syndrome” [3], an underestimation of population abundance in periods prior to official fisheries statistics that should be taken into consideration in the management of natural resources.

Humans inhabited the Baltic Sea basin for millennia, and fishing has always been important. Over time, this ecosystem has experienced drastic anthropogenic changes, but only very recently has the history of these changes been studied [4]–[8]. Continued investigations are essential for drawing a more comprehensive picture of the history of the Baltic Sea ecosystem, especially since most research has covered only part of it. In particular, few studies have examined the easternmost arm of the Baltic Sea – the Gulf of Finland – which is the focus of this research.

**Figure 1:**
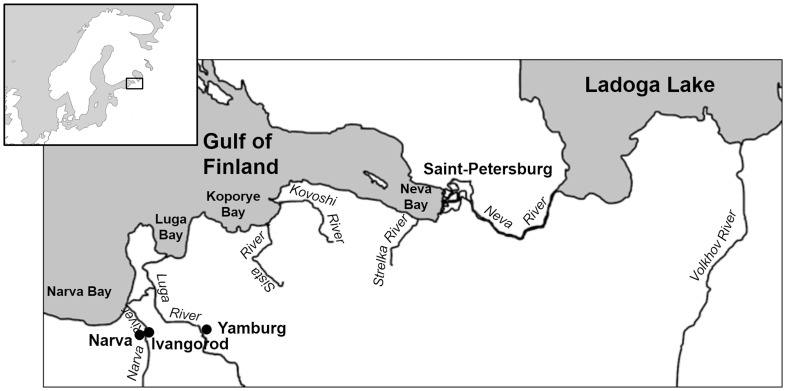
Site map of area where historical datasets on Russian fisheries were obtained.

Bordered by Finland, Russia and Estonia, the Gulf of Finland is a shallow arm intruding inland from the Baltic’s eastern shore. It is frozen from December to March. The Neva River, by far the largest river flowing into the Baltic Sea (average annual discharge is 2500 m^3^/s), causes very low water salinity in the eastern part of the Gulf between the Neva Estuary and Kotlin Island. This shallow water area is called Neva Bay or the Marquis’s Pool. In 2011 it was separated from the rest of the Gulf of Finland by the St. Petersburg Dam. Due to its very low salinity, the fish fauna of Neva Bay is the same as in the Neva River. Salinity of the Gulf of Finland increases westwards but is still rather low (up to 6–7 ppm) near the western border of Russia [9]. The salinity gradient determines fish distribution: only freshwater and migrating species are found in the eastern part, with marine species appearing in the western part of the Gulf. The relatively large Narva and Luga Rivers (discharge 399 and 93 m^3^/s respectively) flow into Narva and Luga Bays in the southwestern part of the Gulf of Finland. There are also several smaller rivers flowing into Koporye Bay for which historical fishery information is available (Fig. 1).

The eastern part of the Baltic has belonged to Russia since the Middle Ages. This territory was controlled by the Great Novgorod and then by the Russian State until it was temporarily conquered by Sweden in the early 17^th^ century. A hundred years later Peter the Great put the area once more under the control of the Russian Empire. Fisheries in the eastern Baltic Sea and its basin have existed since ancient times and were always an important source of food and income. In some settlements professional fishermen constituted a large proportion of the population: the town of Oreshek on the upper stream of the Neva River in the 16^th^ century [10], and the village of Rybatskoe on the middle stream of the Neva River in the 18–19^th^ centuries [11] are two examples. At the same time, fisheries in this region did not play as important a role in the economy as they did in the Russian North [12]–[15]. Most fish products from the eastern Baltic Sea and basin were distributed within the region. Except for lamprey *Lampetra fluviatilus*, few species are known to have had wider Russian or international markets [16].

Early descriptions of the fisheries since the end of the 15^th^ century report a domination of inshore fisheries. By the second half of the 20^th^ century, however, fishing techniques changed dramatically and fisheries moved offshore. Therefore fisheries in the area underwent considerable changes during the last several centuries. The main goal of this study was to trace major trends in development of Russian fisheries and long-term changes in the abundance of key commercial species in the eastern part of the Gulf of Finland. The analysis is based on original archival sources and historical documents new to the literature on fish biology. Making these data available improves historical time series and thus facilitates applying analytical methods in the future.

## Materials and Methods

The Eastern Baltic area passed through many hands during the last several centuries, and historians have had to analyze documents from many nations to create a comprehensive history of fisheries in that region. Data for this study were extracted from historical Russian sources such as cadastres (scribe books) and cadastre-like documents, accounting books, statistical documents, published reports and scientific papers found in the following archives: the Russian State Archives of Ancient Documents (RGADA), the Central Historical Archives of St. Petersburg (CGIA SPb), and the St. Petersburg Branch of the Archives of the Russian Academy of Sciences (PFA RAN).

Cadastres were the principal tax documents recording all taxable economic activities and describing each district village by village. Every farm bore the name of its owner, and a description of arable lands and fisheries was provided. Since the 19^th^ century historians have used this detailed information to create a general picture of local and regional economy in the 16^th^ and 17^th^ centuries.

Scientists have expressed an interest in the fishes and fisheries of the region since the second half of the 19^th^ century. Among them were well-known biologists such as Karl Kessler, Nikolai Danilevsky and Oscar Grimm, whose works are cited in this paper. The first comprehensive fisheries survey in the St. Petersburg region was carried out in 1876–77 by the St. Petersburg Statistical Committee (CGIA SPb, coll. 260, inv. 2, f. 6). Collecting statistical fisheries data had already begun in other parts of Russia, for instance, in the Russian North where the Arkhangelsk Statistical Committee began data collection in 1872 [11]. However, in the St. Petersburg region this initiative was abandoned and catch data were not published, although the numbers of fishermen and fishing income appeared in a multivolume edition on the demographic statistics of the St. Petersburg region [17]. One reason for discontinuation may have been the fragmented data the survey provided [18]. However, even the incomplete data provide valuable information for historical analyses of the fisheries during this period.

A complicated ownership system in a region where free peasant fisheries coexisted with private fishing grounds impeded data collection. The St. Petersburg regional survey (CGIA SPb, coll. 260, inv. 2, f. 6) described only fisheries carried out by local peasant-fishermen, excluding most of the private fisheries. Thus, it accounted for only part of the catch in the area. Additionally during this period, seasonal fishermen, primarily from the towns of Ostashkov on Seliger Lake and Gdov on Lake Peipsi, actively fished in the Gulf of Finland. For instance, in 1889 observers described extremely large nets up to 600–800 m in length that crossed many small tributaries at the mouth of the Neva River and belonged to seasonal fishermen [19]. In the early 20^th^ century the number of seasonal fishermen operating from April to mid-October along the coast of the Gulf of Finland was also large. For instance, about 80 fishermen, who occupied a seasonal village on the southern coast of Neva Bay, primarily used small mesh beach seines and their income was evaluated as “quite substantial” [15].

Estimates of catches from the 15^th^–17^th^ centuries were based on tax records. Payments for harvests from fishing grounds were more often collected in kind, and taxes likely corresponded with tithes, usually a tenth of the harvest, the most common fisheries tax in 16^th^–17^th^ century Russia [11]. We assumed that the fish species taxed in a location were caught locally. Actual catches were calculated as taxes multiplied by ten.

Catch quantities were reported in different units in different sources: numbers of fish, poods (an old Russian unit equal to 16.38 kg) and barrels. Therefore we standardized catches whenever possible. For calculating the average weight of fish we used data from the St. Petersburg regional survey (CGIA SPb, coll. 260, inv. 2, f. 6), which in several cases provided prices for the same species in both weight and numbers. For instance, the cost of one pood of roach was 160 kopecks, and the cost of 100 roach was 100 kopecks. Therefore, about 160 roach made a pood, with an average fish weight of 0.1 kg. In other cases average fish weight was calculated using length data provided by Kessler [20] for the St. Petersburg region and transformed using length-weight relationships (www.fishbase.org).

The size of fish barrels (*bochka* or *kad’* in Russian) varied. Kuratov [21] wrote that salted fish was sold in barrels of different sizes” (p.19). Schultz [22] indicated that barrel size for salted fish ranged from 160 to 320 kg. In our calculations we used the average of these figures, i.e. 240 kg.

## Results and Discussion

### Neva River and Neva Bay

Fisheries data from 11 settlements at the mouth of the Neva River were first reported in the tax records of 1476 [23]. No fishing gear was listed, but the mention of fishing stations provided evidence of regular fisheries. Fishing stations (*tonya* in Russian) are the locations on rivers, lakes or the sea, convenient for fishing, where it regularly took place. Household buildings and other facilities for fishing may sometimes be present in the fishing stations. *Sig*, i.e. whitefish (*Coregonus lavaretus*), appeared in the taxes of all the settlements. A settlement caught 1214 whitefish on average (ranging from 500 to 2000) in 1476… In a few cases catches were provided in numbers and barrels; our calculations assumed 200 fish per barrel, given a standard barrel weight of 240 kg and an average weight of 1.2 kg per whitefish [19].

The scribe book of 1501 [23] mentions 16 villages in the same area that paid taxes out of fisheries, nine of which were the same as in 1476. Fishing gear was not mentioned here either, but eight fishing stations appeared. Whitefish were listed in the taxes of 14 settlements, with an estimated average equal to 996 (from 200 to 2000 fish per settlement). Two settlements only listed *lokh*, or Atlantic salmon (*Salmo salar*). In modern Russian, *lokh* means post-spawning migrants, kelts, but in the earlier times the term was generally used for anadromous salmon [24].

Seventeenth century data exist on fish exported to Sweden via Nyenskans, a port at the mouth of the Neva River where St. Petersburg is located today. Twenty barrels (4.8 mt) of salmon and twenty-four barrels (5.8 mt) of other fish were exported in 1641. The next year thirty-six barrels (8.6 mt) of salmon were exported ([25], pp. 168, 169), however the origin of these fish is unknown.

After 1703, the development of Neva River fisheries depended upon the founding and subsequent rapid growth of St. Petersburg, the new capital of the Russian Empire at the mouth of the river. During the 18^th^ century population quickly grew in the entire region due to the development of St. Petersburg [26], and fisheries rapidly expanded. By 1831 the city listed 38 fishing stations at the river mouth [11], but in 1850 the number of stations had decreased to 15 [27]. Eleven appeared in 1876–1877 (CGIA SPb, coll. 260, inv. 2, f. 6) and the number dropped to 6 before World War I [27]. The St. Petersburg regional survey (CGIA SPb, coll. 260, inv. 2, f. 6) listed 283 fishermen operating in the Neva River. In the early 1880s, 229 local and 120 seasonal fishermen were mentioned [17], and by 1934, the number of local fishermen had increased to 503 [28].

Historically, salmon were extremely important in Neva River fisheries. The first quantitative catch data appeared for 1835–1840, when the source reports that daily catches of salmon approached up to 1,000 fish weighing from 8 to 15 kg each [29]. Unfortunately, records don’t show how many such successful days occurred in each season and no data are provided for other days so estimating yearly catch is problematic. Assessing total salmon catch is possible for the mid-19^th^ century, however, based on calculations from military statistics [27]. This source also lists the total value of St. Petersburg fisheries as 7000 rubles and the price for one whitefish as 0.2 ruble. Using these data we estimated that 2500 salmon were taken near St. Petersburg. Our calculations employed the following assumptions: salmon comprise 70% of this total (from Danilevsky’s observations [30]); salmon were 1.5–2 times more expensive than whitefish in terms of prices per unit weight [11]; the average weight of whitefish was 1.2 kg [19] and the average weight of salmon was 8 kg.

Danilevsky [30] estimated that the value of St. Petersburg fisheries varied in the early 1870s from 40,000 to 80,000 rubles, with salmon as the most important fish. Assuming that salmon provide 70% of the catch and that their price was around 10 rubles per pood (CGIA SPb, coll. 260, inv. 2, f. 6), catches likely ranged from 3500 to 7000 salmon per year, very close to figures obtained when annual salmon catch was about 50 mt, or 6000 fish (total catch given in both numbers and weight: 2975 fish and 1450 poods) (Table 1). In 1934, however, catch had fallen by 44% (Table 2). Interestingly, the size of several salmon populations in the Gulf of Bothnia was relatively stable over most of the 19^th^ century, but had begun to decline by the end of the century [31], [32].

**Table 1:** Annual catches in the southeastern part of the Gulf of Finland by species in the 1870s (CGIA SPb, coll. 260, inv. 2, f. 6) (transformations from number to weight were done using historical data on weight and length [19], [44]; length was transformed to weight using weight-length relationships for the closest available locations).

Species	Average weight, kg	Catches by area, mt					
		Neva River	Luga River	Narva River	Neva Bay	Between Neva andKoporye Bays	Koporye,Luga andNarva Bays
Atlantic salmon *Salmo salar*	8	47.55	1.73	13.00			
Whitefish *Coregonus lavaretus*	1.3	8.71					5.2
Smelt *Osmerus eperlanus*	0.033	90.97	0.12		0.66		
Vendace *Coregonus albula*	0.04	54.93			0.20		
Lamprey *Lampetra fluviatilis*	0.061		2.99	0.09			0.36
Vimba bream *Vimba vimba*	0.333		1.71	0.33	8.33	16.65	0.78
Twaite shad *Alosa fallax*	0.3		0.04				
Roach *Rutilus rutilus*	0.1	0.02	2.69		16.65	5.00	0.49
Ruffe *Gymnocephalus cernuus*	0.05	0.15	0.16		8.95	2.50	0.44
Bream *Abramis brama*	0.47	0.08			78.86	23.50	
Perch *Perca fluviatilis*	0.07	0.21	1.97	0.49	11.69	3.50	0.25
Pike *Esox lucius*	1	0.18	1.55	0.84	0.50		0.44
Ide *Leuciscus idus*	1.2	0.21	2.64	0.16	0.25		0.44
Burbot *Lota lota*	0.5		0.86	0.29			
Pikeperch *Sander lucioperca*	0.887	0.04		0.33	125.07		
Brown trout *Salmo trutta*	0.4		0.08				
Grayling *Thymallus thymallus*	0.3		0.03				
Bleak *Alburnus alburnus*	0.008		0.08				
Herring *Clupea harengus*	0.024				0.36		196.56

**Table 2:** Catches in Neva River in 1934 (mt) [27] (presented catch sizes for the Neva River, according to author’s estimate, comprise approximately 70% of actual catch because they do not account for personal consumption and non-professional catches).

Fish name	Catch
Atlantic salmon	26.7
Whitefish	2.6
Smelt	561.0
Lamprey	53.6
Roach	13.2
Ruffe	11.2
Whitefish	2.6
Bream	1.7
Bleak, pike, burbot, eel	1.4
Others	29.8

Decreases in catch were also observed for whitefish from the 1870s to 1934 (Table 1 and 2). This was evident for salmon and whitefish despite an increase in fishing effort. Thus, the two most important commercial fish populations and fisheries prosecuted in the Neva River since the 15^th^ century gradually lost their significance. Overfishing and pollution likely contributed to the decline of salmon and whitefish. Climate changes were likely not as important because climate in the 1930s is known to have been quite warm. Our studies of salmon in the Russian North have shown that salmon populations were more abundant in warmer periods [33].

In 1876–1877 salmon still provided the greatest economic value in comparison with other species, but the highest catches in that period were of *koriukh* (smelt *Osmerus eperlanus*). Soon, however, smelt exceeded salmon in economic value due to further growth in their catch. Fishing effort increased in the 1920–30s to meet increased demand for protein in the USSR. However, even in 1934, smelt catches were probably far below their potential because catches rose to 10 times the 1934 level between 1946 and 1995 [34].

The most considerable changes in the Neva River between the 1870s and 1930s occurred with vendace *Coregonus albula*, lamprey and flounder *Pleuronectes flesus*. Vendace fisheries were very important in the 1870s [30], but were not even mentioned in 1934. The opposite is true of lamprey, which were absent in the St. Petersburg regional survey (CGIA SPb, coll. 260, inv. 2, f. 6), but became very important in the 1930s, with catch estimated at about 60 mt (1931) [35]. Vendace became important again after World War II, with an annual average catch of about 80 mt for the period 1946–1960, similar to catches in the 1870s, although maximum catch exceeded 1000 mt in 1959 [36]. Lamprey catches were quite stable after World War II (average annual catch, 16.6 mt), however, at a notably lower level than in 1934 (77 mt). Later 20^th^ century catches usually fell between 20–90 mt [36]. Flounder were not listed in Neva River and Bay catches in the 1870s and 1930s, although narrative accounts describe abundant populations before 1910 [37]. We assume natural fluctuations, most likely caused by changes of salinity or temperature, occurred in these three species before the 1930s. Their commercial value was no as high as that of Atlantic salmon or whitefish. Thus we believe that fishing pressure on their populations was comparatively low and did not encourage overfishing, although pollution could have been a factor in changing abundance. Comparing Neva River catches in the 1870s and 1930s shows that total catch of these species increased more than 3 fold (Tables 1 and 2), and suggests that 1870s fisheries were probably far from their potential.

Among commercial fish species, a special place belongs to the Atlantic sturgeon *Acipenser oxyrinchus,* which replaced the native *A. sturio* in the Baltic Sea about a thousand years ago [38]. Sturgeon migrate from the Baltic Sea through the Neva River and Ladoga Lake to spawn in the Volkhov River. According to archeological excavations begun in 1911–13 and continuing today, intensive sturgeon fisheries existed in the lower course of the Volkhov River as early as the 8^th^–9^th^ centuries. Analysis of bone remains show that sturgeon ranged from 1.7–3.1 m in length and 100–180 kg in weight [39]. The scribe book for 1569 reported sturgeon fisheries in the mouth of the Volkhov River, near the town of Ladoga, carried out by 54 fishermen using 27 nets called *poezd (* [1]0, p. 152). *Poezd,* a small (4–6 m long) drift net operated from two boats [15], was a common gear in northern Russia.

As late as the end of the 18^th^ century regular sturgeon fisheries still took place in the Volkhov River [40], [41], however by the mid-19^th^ century catches were described as insignificant (PFA RAN, coll. 129, inv. 1, f. 496, l. 1). Several sturgeon were caught each year in the river before the Volkhov Hydropower Dam was constructed in 1926 [41]. Even as late as the 1960s annual catches comprised 200–300 kg, according to official statistics [42]. The last Neva River sturgeon, weighing 26 kg, was caught in Ladoga Lake in 1984 [43].

Reports of large sturgeon in the Neva River exist for the 19^th^ century: Kessler [19] described a 215 kg sturgeon caught in 1851 and another of similar size taken about twenty years earlier. Berg [44] reported two sturgeons weighing 160–180 kg captured during the mid-19^th^ century. Sturgeon occasionally appeared with catches of other species such as eel, lamprey and pikeperch in the mouth of the river ([27], p. 257). As late as 1934 two smaller sturgeons weighing 4 and 96 kg were reported [28], [44].

In the 1870s, freshwater fish such as pikeperch *Sander lucioperca*, bream *Abramis brama*, roach *Rutilus rutilus*, European perch *Perca fluviatilis*, ruffe *Gymnocephalus cernuus*, pike *Esox lucius*, and ide *Leuciscus idus* were caught outside the river in Neva Bay and comprised about half of the total catch in both river and bay (Table 1). By the 1930s freshwater fish catch had decreased by about half, however, these species made up less than 7% of the total due to an intensification of the smelt fisheries (Tables 2 and 3). In general, information on freshwater fish is scarcer than on migrating fish due to their lesser commercial significance.

**Table 3:** Catches in different parts of the Gulf of Finland (mt) (average from 1933 and 1934 [57]).

Fish name	Neva Bay	Between Neva and Koporye Bays	Koporye. Luga and Narva Bays
Atlantic salmon	6.84		39.85
Smelt	1125.22	22.81	137.37
Lamprey	35.13		30.49
Eel	1.73	0.49	23.99
Herring	2.09	42.23	4270.37
Others	73.14	98.26	448.74

In summary, salmon and whitefish have been the most significant commercial species harvested on the Neva River since the 15^th^ century. During the 20^th^ century, however, the salmon fishery never again reached the maximum historical catches of 6000–7000 fish, and gradually lost its commercial significance, even though in some years catch was still quite high, for instance, 36 mt or about 4500 fish in 1949, and 23.2 mt or 2900 fish in 1985 [36]. A similar picture was observed for whitefish. Catch peaked in 1952 at 97 mt [46], but then decreased quickly and the species had disappeared from fishery statistics by the 1990s. Sturgeon lost significance much earlier, although occasional specimens were caught commercially until the 1960s. Since the 2000s smelt have become the most important commercial fish in the Neva River, together with lamprey and vendace. However, these species experience notable long-term fluctuations in their populations. Catches of smelt increased during the second half of the 20^th^ century, but drastically declined in the 1990s [36], and today the commercial significance of the Neva River smelt is low [47].

### Luga River

Historical sources described fisheries in several right tributaries of the Luga River in 1501 [23] (Fig. 1). Records documented four settlements fishing for whitefish, smelt, *losos’*, and *belaya ryba.* The two latter names need special consideration. In modern Russian, *losos’* usually means Atlantic salmon, but in older records it may also mean brown trout *S. trutta*, which have a similar appearance. There is the possibility that fishermen did not distinguish between the two species, so we used the generic term “salmonids” in our translation. *Belaya ryba* literally translates from the Russian as “white fish”, a generic name for fish of white color and therefore potentially referring to several cyprinid species such as vimba bream, roach, dace *Leuciscus leuciscus,* ide or the whitefish *C. lavaretus* usually called *sig.* For gear, sources indicate that only weirs were used, but the size of catches was not provided. Also, in 1500–1501, fisheries were recorded in two settlements on the Luga River near the town of Yam (later known as Yamburg, currently Kingisepp), and inhabitants paid tithes from their catches. Weirs were located on a right tributary of the downstream part of the Luga River [48].

Russian sources described a well-developed fisheries infrastructure in the Luga River in the early 18^th^ century. This area belonged to Sweden from 1617 to 1703 and the Swedish administration likely paid much attention to fisheries development there. Weirs, including a type known later as *Koza,* were set up in the river near Yamburg (PFA RAN, coll. 129, inv. 1, f. 495, 4980); [24]. Information on fish sold at the Luga town market is presented in Table 4, however, we don’t know what part of the total catch these figures comprise. They characterize the relative importance of different species in culture and society rather than the total size of the catch in the river.

**Table 4:** Fish (numbers) sold at market of Luga town in 1730 and 1732 (RGADA. coll. 1239. inv. 2. f. 588. 690).

Species	Year 1730	Year 1732
Atlantic salmon	267	273
Brown trout	155	194
Whitefish	605	226
Vimba bream	125	90
Lamprey	3600	1100
Burbot	255	155
Pike	10	
Ide	10	

As on the Neva River, Atlantic salmon supported the most important local fisheries and were also common cultural symbols: for instance, the town of Luga in 1781 adopted an emblem decorated with a salmon in a tub. But in 1851 Karl Ernst von Baer reported that very few salmon were caught near Yamburg due to extensive fisheries located at the Luga River mouth ([49], p. 11–12). During the mid-19^th^ century, Baer inspected Luga River fisheries as head of the Baltic Expedition organized by the Ministry of the State Domain [50]. As upstream salmon fisheries failed, *Koza* lost its importance and was gradually abandoned. The St. Petersburg regional survey (CGIA SPb, coll. 260, inv. 2, f. 6) reported 45 fishermen in eight villages on the Luga River near Yamburg. The downstream part of the Luga was fished more actively, with 729 peasants engaged in fishing, although some likely fished part time. Total catches on the Luga River are shown in Table 1. Lamprey provided the greatest catch. Unfortunately, the survey data only reflect peasant catches, while the most important fisheries in this area were private. Nevertheless, this provides minimum harvests of Atlantic salmon and lamprey and indicates the local importance of these fisheries.

Similar to previous periods, Grimm [24] reported intensive fishing in the river in 1889, chiefly targeting Atlantic salmon. Fishing for salmon started in Luga Bay. Several traps were set up in the mouth of the river, upstream from sites where beach seines were used. According to Grimm’s estimates, about 80% of the spawners were caught by these gear. Twenty more salmon traps and many nets were located even farther upstream from the beach seining location. Finally the last *Koza* and another weir were situated near the spawning grounds. On the spawning grounds salmon were also fished by harpoons and drag nets.

Despite such intensive fishing, catches persisted at high levels during the next several decades. Grimm estimated annual salmon catch as 2000–5000 fish at the late 1880s. At the turn of the 20^th^ century, catch rose to 10,000 fish, but this may have been influenced by the operation of hatcheries starting in 1893–1894 [51]–[53]. After the hatcheries closed in 1912, catches leveled off at 3000–3500 fish in 1929–1934 [54], until dam construction near Kingisepp (formerly Yamburg) contributed to a drastic population decline. Currently, the population is supported by hatchery stock [53], although some natural reproduction still exists [55].

A decrease similar to that of Atlantic salmon also occurred in the whitefish population. Grimm [24] wrote that these fish were very numerous in the Luga River before timber rafting. Data for the 1730s show that the amount of whitefish caught was quite large in comparison with other fish. But they do not appear in the St. Petersburg regional survey (CGIA SPb, coll. 260, inv. 2, f. 6). Among factors responsible for the decline of salmon and whitefish populations, Grimm mentioned timber rafting and coniferous branches used in weir construction that were left in the rivers after the weirs were destroyed. However, the mechanisms inducing population decline are unclear, or Grimm may have overestimated their effect since the damage took place downstream from spawning grounds.

By the end of the 1880s, vimba bream and lamprey joined Atlantic salmon as the primary commercial fish on the Luga River. Grimm [24] evaluated catches of vimba bream as 5000–6000 fish per year (the St. Petersburg regional survey (CGIA SPb, coll. 260, inv. 2, f. 6) gives a figure of 5146 fish). The amount of lamprey caught was not reported, but circumstances suggest that it was large. In autumn during lamprey migration, almost all peasants fished for them, and one day’s *morda* fishing usually brought in 40–1000 fish ([17], pp. 204–205). Thus, a total catch of about 50,000 lamprey with a total weight of 3.0 mt, figures reported by the survey, probably underestimated actual catches. In total, Grimm [24] listed 34 fish species in the Luga River, 27 of which were fished; yet by the late 1800s sturgeon rarely entered the river.

In two districts in the downstream part of the Luga River, the St. Petersburg regional survey (CGIA SPb, coll. 260, inv. 2, f. 6) mentions a fish named *faler*, caught in small quantities of 20 and 100 fish. We were not able to identify this fish. Two options seem most probable. Today, twaite shad *Alosa fallax* are distributed as far east as Luga Bay [45], but evidence of spawning grounds in the Luga river is absent. If *faler* are twaite shad, the survey information extends their former distribution eastward into the Gulf of Finland. *Faler* may also be a misspelling of the Russian name for brown trout, *forel*. Freshwater fish contributed 60% of the total catch in the 1870s; roach, ide, perch, pike and burbot were the most important.

Thus, fisheries in the Luga River focused primarily on Atlantic salmon since earliest times, and on whitefish later, before their decline at the end of the 19^th^ – first third of the 20^th^ century due to overfishing and dam construction. Significant fisheries also targeted lamprey and vimba bream. About half of the total catch was likely comprised of freshwater species with lower commercial value.

### Narva River

Fisheries in the Narva River go back to at least 1240 on the western shore in the town of Narva, which belonged to Denmark at that time. Interestingly, sturgeon appeared on Narva insignia in the 14–15^th^ century, but this design was apparently abandoned later on. This may signal a serious decline in the sturgeon population after 1500. Russian fisheries in the Narva River started with the founding of the town of Ivangorod in 1492 on the eastern bank of the river. The inhabitants of Ivangorod and adjacent villages operated weirs in the 15^th^–16^th^ centuries, the numbers of which were: 30 weirs in 1499 [56], 55 weirs in 1565 (32 big and 23 small), and 39 weirs in 1572 (20 big and 19 small) (RGADA, coll. 137, inv. 1, f. 8). Sources listing “Baltic herring, roach and other fish” as targets [56] indicate that fishing took place not only in the river, but also at sea. Wide use of weirs suggests that Atlantic salmon were very important, although sources contain no direct proof of this.

In 1851, significant eel fisheries were described in the upstream section of the Narva River near the village of Skamya (PFA RAN, coll. 129, inv. 1, f. 494). The annual catch brought in 4,000 silver rubles per year, and the price of 100 eels varied from 30 to 40 rubles. Thus the total annual eel catch can be estimated as 10,000 fish. Peasants used a special gear in this fishery, called a *stozh*. Each *stozh* carried twenty sack-like traps. Traditionally, each fisherman used eight *stozh’es* but by 1851 that number had dropped to three.

The value of downstream Narva River fisheries in the villages of Popovka, Sarkulya (Sarkul’) and Venkulya (Venkul’) was even larger than that of upstream fisheries; total catch was 6,000 silver rubles per year and salmon was the main targeted species ([27], pp. 257–264). According to the St. Petersburg regional survey (CGIA SPb, coll. 260, inv. 2, f. 6), salmon catch of 1625 fish (13.0 mt) comprised 84% of the total value of Narva River fish. The rest were chiefly freshwater fish, and eel were not even mentioned in the survey. However, this could be due to the fact that the survey dealt only with downstream fisheries, whereas eel were fished upstream. Salmon fisheries in the Narva River existed until the mid-20^th^ century, with maximum catches occurring in that period. For instance, in 1948, catch varied from 18 to 25.2 mt according to different sources [53]. But after construction of the hydroelectric power station in the early 1950s, salmon became extinct. Later the Narva River was re-populated with salmon from other rivers (the Neva, Daugava, Gauia and Luga) [57]. Narva River freshwater species comprised only about 14% of the total catch in the 1870s, with pike as the most important fish, considerably lower than on the Luga and Neva Rivers.

Therefore, Narva River Atlantic salmon harvests of 2000–3000 fish persisted until the extinction of the sub-species in the mid-20^th^ century due to dam construction. The role of overfishing in their disappearance is unclear. This river was probably fished more intensively than the Neva and Luga Rivers during the period when the region was under Danish and then Swedish authorities. This caused the disappearance of sturgeon in Middle Ages. In some periods, noticeably the mid-19^th^ century, eels were preeminent in Narva River fisheries, producing at least several thousand fish annually.

### Small Rivers Flowing into Koporye Bay

In 1470 and 1501, fisheries existed in 12 settlements along the Sista, Strelna, Kovosha (now Kovoshi) and Kernova (Voronka) Rivers flowing into Koporye Bay [23] (Fig. 1.). The following fish were presented in tax records for this period: smelt, whitefish, salmonids (Atlantic salmon, brown trout or both), and cyprinids. Smelt were mentioned in five settlements in 1470 and in two settlements in 1501. Catch estimates for this species ranged from 3,000 to 10,000 fish (100–300 kg). Whitefish were mentioned, with catches of 10 barrels per village (about 2.4 mt) in one village in 1470 and in two villages in 1501. Salmonids caught at the rate of 20–30 fish per village were recorded in one village in 1470 and in three villages in 1501.

Notable changes in fishing gear occurred in this area between 1470 and 1501, most importantly, the appearance of weirs. Because of the construction and maintenance effort involved, their appearance in 1501 probably indicates an increase in the economic significance of fisheries in the area. With weirs, catches of valuable salmonids grew considerably, and smelt replaced salmon for taxation purposes. Sources do not record the type of fishing gear used before weirs, but beach seines were traditional for Russians, and even baskets were suitable for catching smelt during their upstream spawning migration.

**Figure 2:**
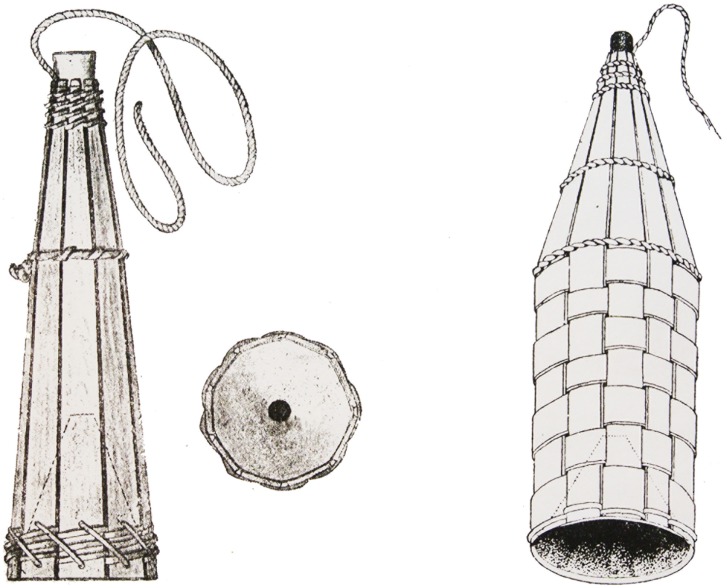
Traps for fishing lamprey in the Neva River: *burak* (left) and *korzina* (right) (from [35]).

Eighteenth-century state fishery account books report that the following fish were sold in the Koporye town market: salmonids, whitefish, lamprey, Baltic herring and vimba bream (RGADA, coll. 1239, inv. 2, f. 670, l. 20 verso; f. 654, l. 11 verso). The presence of herring shows that fisheries had already spread to the sea, and lamprey indicate the use of specialized fishing gear such as “*morda”* or *“burak”*, small cone-like traps made of rods (Fig. 2). Among species caught in Koporye Bay tributaries, the St. Petersburg regional survey (CGIA SPb, coll. 260, inv. 2, f. 6) reported freshwater species such as pike, ide, roach, ruffe and anadromous vimba bream and lamprey. No salmonids or whitefish appear from the rivers, although whitefish were listed among fish caught in Koporye Bay.

Today, Atlantic salmon are absent from the Sista, Kovoshi, Voronka and Strelka Rivers in the southeastern part of the Gulf of Finland [58], although our historical materials document catches of salmon or trout. We assume that the use of weirs in the 16^th^ century probably indicates the presence of a salmon fishery because weirs are the typical gear for fishing Atlantic salmon in Russia [13]. However, these weirs may have contributed to the disappearance of Atlantic salmon so long ago that no direct scientific evidence exists for salmon in these rivers. Unfortunately, historical data cannot ultimately prove that Atlantic salmon populated these rivers in the 15^th^–16^th^ centuries because the terminology can also refer to brown trout, which currently inhabit these rivers, but this possibility should be taken into account when considering restoration programs for this species. It should be also considered that in the past rivers might have provided better environmental conditions for salmon populations because less water was diverted for human use 500 years ago.

In sum, small river fisheries focused on migrating species (smelt, whitefish, vimba bream and lamprey). Salmonids played some role, but it is not known conclusively that Atlantic salmon were present. Freshwater species were probably more important in small rivers than in large rivers, but data on them are quite scarce.

### Eastern Gulf of Finland

Mikhin [59] suggested subdividing the eastern Gulf of Finland into several parts depending upon salinity, a chief determinant of fish community composition: (i) Neva Bay, inhabited mostly by freshwater species; (ii) the region from Neva Bay on the east to Koporye Bay on the west, inhabited by freshwater and marine species; and (iii) Koporye, Luga and Narva Bays, inhabited mostly by marine species with some amount of migrating. We generally follow this division (Table 1), although Neva Bay fisheries were described in the Neva River section.

Earlier sources do not always provide information about fishing locations, but the presence of marine fish such as Baltic herring in the catch inform us that sea fishing had occurred. Herring come as far east as Neva Bay in some years, but the western border of their distribution is usually Koporye Bay. Another important marine species in the Eastern Baltic, sprat *Sprattus sprattus*, were not mentioned in inshore fisheries records at all (although sprat can mix with herring [60]).

Herring fisheries have existed since at least the 16^th^ century, when they were mentioned among the species caught near the mouth of the Narva River [56], however their role before the 1870s is not easy to determine. The infrequent appearance of herring in documentary sources before this period may indicate that they were not as economically important as freshwater and migrating fish.

The St. Petersburg regional survey (CGIA SPb, coll. 260, inv. 2, f. 6) showed extensive development of herring fisheries in the Gulf of Finland (Table 5). Fishing generally took place through ice in the winter; beach seines were employed (usually 10 fishermen per net) and catch was transported by horses and wagons, although horses were not always mentioned in the survey. In warmer seasons fishing took place from boats. In both cases fishing took place 5–20 km from shore. In 1876–1877, 83% of the herring were caught in Luga Bay, with the rest coming from Narva and Koporye Bays.

**Table 5:** Catch and effort in herring fisheries in the eastern Gulf of Finland according to the St. Petersburg regional survey (CGIA SPb. coll. 260. inv. 2. f. 6).

Location	Fishermen	Means of transportation	Catch. number
Neva Bay	80	38 boats	15000
Between Neva and Koporye Bays	37	16 boats	
Koporye Bay	88	13 boats	530000
Luga Bay	580	49 boats, 108 horses	6837500
Narva Bay	120	18 boats	822500

Data on Soviet fisheries in the Gulf of Finland in the 1930s [59], [61] reported considerable increase in herring catches. By 1933–1934 the total catch of herring increased 17.5 times in comparison with 1876–1877 amounts (assuming an average weight of fish = 30 g), largely due to increased catches in Koporye, Luga and Narva Bays, rather than in Neva Bay. During this period the number of fishermen rose from about 1600 [17] in the 1870s (CGIA SPb, coll. 260, inv. 2, f. 6) to 7214 in 1931 [61], however, the number of fisherman per boat stayed roughly the same (6,7 in 1870s and 7,7 in 1831). Therefore catch per unit effort increased 4-fold during 5 or 6 decades, perhaps in consequence of greater herring abundance or improved fishing methods.

Mikhin and Antipova [61] reported the presence of several marine species with no commercial value in the western part of the study area: sprat, fourhorn sculpin *Tryglopsis quadricornis*, twaite shad *Alosa finta*, cod *Gadus morhua*, eelpout *Zoarces viviparus*, flatfish *Pleuronectes flesus*, sticklebacks *Gasterosteus aculeatus* and *Pungitius pungitius*, lumpsucker *Cyclopterus lumpus.* Thus, Baltic herring was the only commercially fished marine species in the region. Herring fisheries had been mentioned in sources since the 16^th^ century, but for long periods of time the fisheries exploited only a small portion of herring potential in the region. In the 1870s total herring catch was about the same as the total catch of all other species in the region combined. Later on, regional fishing effort developed and grew much faster for herring than for migrating and freshwater species. That herring populations had higher productivity potential is shown by subsequent catches: Baltic herring catch in the 1960–90s exceeded that in the 1930s more than three-fold [36].

## Conclusions

In this study, we report new data extracted from archival sources and historical literature related to fisheries in the Eastern Gulf of Finland from the 15 to early 20^th^ centuries. We put them in biological context to facilitate usage by fish biologists and policy makers.

Fishing gear notably developed during the study period. In the early cadastres weirs and drift nets were most frequently mentioned. This gear combination is well known in other regions of Russia. In addition to being very effective individually, weirs facilitate the use of gill nets because fish are concentrated in front of the weir. Although a primitive technology, weir construction and maintenance take much effort. Historically they were constructed of branches, often of fir. In the mid-19^th^ century weirs started to be replaced by larger traps. Beach seines were also common in river and inshore fisheries. Later on, fixed nets appeared and were deployed under the ice in the winter herring fisheries. Specialized gear was used for capturing eel (*stozh*) and lamprey (*morda*).

In this study we analyzed the dynamics of fish populations using catch data. Catch per unit effort (CPUE) would have provided a better proxy for population abundance because it accounts for fishing effort, but data on fishing effort are rare in old records. In addition, this study mostly operates with catches of anadromous fish in rivers. Riverine gear, which usually do not change much in construction and are used in the same locations over centuries, exhibit much more stable fishing effort than gear deployed at sea.

Although fragmentary, the data allow us to trace long-term trends in fisheries patterns and fish populations. Such trends are sometimes difficult to comprehend in short-term data series. One clear pattern is the gradual movement of major fishing locations from upstream to downstream river sections. We suspect that the decrease in the number of fishing weirs from 55 to 39 near Ivangorod on the Narva River from 1565 to 1572 was the first indication of this trend. By the 19^th^ century the main fishing areas on both the Luga and the Narva Rivers (excluding fisheries for catadromous eels) were located near their mouths. The trend was also visible in Neva River fisheries, as catches in Neva Bay increased much more between the 1870s and 1930s than catches in River itself. However, this move took place later than in the Luga and Narva Rivers likely because the Neva is larger.

The shift from river to marine fisheries is typical of fisheries development in general, and occurred in many parts of Western Europe almost a millennium ago. Mostly, it resulted from the overfishing of freshwater and diadromous species, but it was also facilitated by river pollution due to industrial development and population growth [62], [63]. Barret and coauthors [64] showed that marine fishes like cod started to dominate in the diet of West Europeans after the 10–11^th^ centuries. Historically, diadromous sturgeons, followed by salmon, were the first species that declined [62], [65]. This phenomenon was reported for the most densely populated parts of Europe, including the Southern Baltic area.

In the eastern Baltic Sea this shift took place much later, in the 19^th^ century. Today no commercial fisheries are located in rivers except a traditional lamprey fishery that inhabitants of St.Petersburg conduct in autumn from bridges crossing the Neva River. Sources clearly show that other trends, such as a progressive development of fishing gear and a growing intensity of fishing effort, resulted in considerably increased pressure on fish populations. The effect of fishing pressure was compounded by habitat degradation: pollution, dam construction etc.

Changes in fishing patterns are closely and mutually interrelated with changes in fish abundance: changing fishing patterns can cause, and are affected by, changes in fish abundance. In the eastern Gulf of Finland, the movement of fisheries downstream was caused in part by competition among fishermen for better fishing locations. Given that the main target species were anadromous, downstream fishing sites had obvious advantages, especially with declining populations. At the same time, increased fishing pressure at the mouths of rivers resulted in faster declines of anadromous fish populations.

Historically, the first overfished species in the Gulf was sturgeon, now more endangered than any other group of species worldwide (http://www.iucn.org/?4928). Precious roe and meat combined with slow growth and maturation, a comparatively simple fishing technique, and vulnerability to habitat degradation and pollution resulted in the extinction of sturgeon in the Baltic Sea. Historical evidence for sturgeon fisheries is scarce because the majority of river sturgeon populations declined long ago. However, archeological evidence indicates that sturgeon were among the earliest target species in the area. For instance, in the 8^th^ century sturgeon were already important food fish in the town of Staraya Ladoga on the Volkhov River near their spawning grounds [66]. Most likely, the Neva river sturgeon population became extinct later than populations in other Baltic rivers. It maintained commercial significance until the 1960s. The last known sturgeon in the Baltic Sea was caught in 1996 near Saaremaa Island [67].

Other migrating fish such as Atlantic salmon, whitefish, smelt and, to a lesser extent, vimba bream were the basis for regional fisheries for most of the study period. Except for smelt, these species had experienced a notable decline by the early 20^th^ century and have now completely lost commercial significance. Smelt maintained significance longer due to higher abundance until very recent times, but now Neva River smelt have experienced a drastic decline caused not only by overfishing but also degradation of their spawning grounds [47]. Current efforts promoting smelt as part of St. Petersburg’s cultural and gastronomical heritage have sound historical basis, but conservation measures may be inadequate to maintain sufficient resources. Eel and lamprey appear in Russian sources quite late, only in the 18^th^ century, probably because they required specialized fishing gear, or due to market changes. Eels were important in the mid-19^th^ century in the Narva River, but then declined. Lamprey, although less important historically than salmon, whitefish or smelt, have remained commercially viable at present, probably due to limited demand. Fluctuations of the anadromous lamprey and vendace show no declining trend. Unlike other migrating fish, their abundance may be driven by natural factors.

Freshwater species such as roach, ide, pike, perch, ruffe, burbot regularly occur in historical sources. Although in some areas they exceeded half of the total catch, data on these fish is poor because their commercial value was generally low. They were not as important as migrating fish and do not show any definite trends in abundance.
